# A temperature-adaptive logit-standardized knowledge distillation for lightweight network intrusion detection

**DOI:** 10.1371/journal.pone.0361860

**Published:** 2026-11-02

**Authors:** XiaoFang Dong, Kai Yang, JingChao Liu, Wen Chen

**Affiliations:** 1 School of Computer, Xijing University, Xi’an, Shaanxi, China; 2 Xi’an Key Laboratory of Human–Machine Integration and Control Technology for Intelligent Rehabilitation, School of Computer Science, Xijing University, Xi’an, Shaanxi, China; Nanjing Forestry University, CHINA

## Abstract

The rapid growth of the Internet has made network attacks increasingly frequent, stealthy, and diverse, posing stringent requirements on the accuracy and latency of network intrusion detection systems (NIDS). Although deep-learning-based NIDS can achieve strong detection performance, their large parameter size and high computational cost hinder deployment on resource-constrained edge devices. To address this limitation, we propose a temperature-adaptive logit-standardized knowledge distillation framework for lightweight network intrusion detection. A high-capacity teacher network first learns discriminative representations from network traffic, and a compact student network is then trained to mimic the teacher under a tailored distillation scheme. During distillation, we apply per-sample Z-score standardization to the logits of both teacher and student models to mitigate scale mismatch and emphasize relative inter-class relationships. Moreover, we adapt the distillation temperature according to sample difficulty and training progress, enabling suitably smoothed soft targets across samples and stages, and jointly optimize the student with hard-label supervision. Experiments on benchmark datasets including NSL-KDD and CIC-IDS2017 demonstrate that the proposed method substantially reduces model complexity while maintaining stable and competitive detection performance. Additional repeated-run, ablation, and lightweight baseline analyses further indicate that the proposed framework achieves a favorable accuracy–complexity trade-off.

## 1 Introduction

With the continuous advancement of informatization, modern networks have become a fundamental backbone for social operation and critical national infrastructures. Meanwhile, cyberattacks have been rapidly evolving–-from conventional port scanning and Denial of Service (DoS) attacks to today’s stealthier and more persistent threats, such as Advanced Persistent Threats (APTs), zero-day exploits, and customized intrusions targeting specific business scenarios. Timely and accurate detection and mitigation of diverse attacks in large-scale, highly dynamic network environments, while ensuring the confidentiality, integrity, and availability of information systems, has thus become a central challenge in cyberspace security. In this context, NIDSs serve as a key component of cyber defense by continuously monitoring and analyzing network traffic or host behaviors to identify malicious activities and raise alerts [1]. However, in practical deployments, NIDS models must operate under massive high-dimensional data, complex traffic patterns, and ever-changing attack behaviors, thereby facing stringent constraints in detection accuracy, real-time responsiveness, and scalability.

In recent years, deep learning has achieved remarkable success in computer vision and natural language processing, and its powerful capability of automatic feature extraction and end-to-end modeling has also been widely adopted for intrusion detection tasks [2]. Representative architectures, such as Convolutional Neural Networks (CNNs), Recurrent Neural Networks (RNNs), and Long Short-Term Memory (LSTM) networks, can learn complex temporal dependencies and spatial distribution patterns from traffic data, and typically outperform rule-based or shallow learning approaches in identifying sophisticated attacks and unknown threats. Nevertheless, these deep models often involve large parameter sizes, high computational complexity, and substantial memory footprint. They rely on high-performance hardware during training and still require considerable processing capability and resources for inference. In particular, for online detection under high concurrency and strict latency constraints, deploying heavyweight deep networks can significantly increase computational and energy costs, hindering long-term stable operation in real-world networks.

With the rapid development of the Internet of Things (IoT), Industrial Internet of Things (IIoT), and edge computing, a large number of end devices and edge nodes are now capable of data collection and on-device computation, making the “device–edge–cloud” collaborative protection paradigm an increasingly important direction for intrusion detection. However, such end/edge devices are typically resource-constrained, featuring limited CPU capability, small memory/storage, and strict power budgets. Directly deploying complex deep intrusion detection models on these platforms often results in excessive inference latency, insufficient throughput, or even failure to fit the model into device memory, which severely limits the practical adoption of high-accuracy NIDS at the edge and on endpoints. Therefore, compressing and lightweighting deep intrusion detection models–-while maintaining detection accuracy and inference efficiency–-has become a key research problem for intelligent NIDS in resource-constrained environments [3].

Prior studies have attempted to improve robustness and accuracy by adopting Generative Adversarial Networks (GANs) for data augmentation and designing multi-channel deep models with spatiotemporal feature fusion. However, these approaches often further increase model complexity and computational burden, leading to higher deployment and maintenance costs in edge scenarios [4]. In contrast, model compression techniques such as Knowledge Distillation (KD) leverage a teacher–student paradigm to transfer the so-called “dark knowledge” from a high-capacity teacher model to a compact student model, which can substantially reduce parameter footprint and computation while retaining the teacher’s discriminative capability to a certain extent [5]. In the context of network intrusion detection, developing lightweight KD-based detectors that preserve (or closely approach) the original detection performance is of considerable theoretical and practical value for improving detection efficiency, reducing deployment cost, and enabling large-scale adoption of intelligent NIDS in edge environments.

Nevertheless, directly applying conventional KD to intrusion detection still faces several challenges. First, the widely used fixed-temperature smoothing strategy during distillation is often unable to accommodate varying sample difficulty and the student’s evolving needs across training stages, which may produce distillation signals that are globally over-soft or over-hard and thus degrade knowledge transfer effectiveness [5,6]. Second, intrusion detection datasets typically exhibit severe class imbalance, and different attack categories may lead to logits with substantially different magnitudes and variances in the teacher model. When the numerical scales of teacher and student logits are misaligned, the distillation loss can be dominated by a few extreme outputs, weakening optimization stability and undermining transfer quality. Hence, there is a pressing need for a refined distillation mechanism that can adaptively adjust the distillation temperature and normalize the logit scale, thereby improving the training stability and detection performance of lightweight NIDS under resource constraints.

To address the above challenges, this work investigates knowledge-distillation-based lightweight intrusion detection and proposes a distillation framework that integrates adaptive temperature scheduling with logit standardization. Specifically, a high-capacity deep fusion model is adopted as the teacher, while a more compact student network is constructed by compressing convolutional channels and reducing fully connected layers, thereby lowering parameter count and computational cost for resource-constrained deployment. During distillation, the temperature is dynamically adjusted according to training stage and sample difficulty to provide more suitable soft-target supervision across samples and epochs. In addition, per-sample Z-score standardization is applied to the logits of both teacher and student models to alleviate scale mismatch and reduce the adverse effect of extreme logits on distribution alignment. By jointly optimizing hard-label supervision and soft-target distillation loss, the proposed framework aims to improve the stability and effectiveness of lightweight intrusion detection under complex and imbalanced data conditions. This design is particularly motivated by the need for a more stable and deployment-oriented KD pipeline in lightweight NIDS scenarios, where both training robustness and inference efficiency are important.

The main contributions of this paper are summarized as follows.

1. We develop a teacher–student lightweight NIDS framework for resource-constrained IoT and edge environments. A high-capacity deep fusion model is used as the teacher, while a compact student model is constructed through systematic compression of convolutional channels and fully connected layers, substantially reducing parameter footprint and theoretical computation;
2. We investigate a lightweight distillation strategy that integrates adaptive temperature scheduling and logit standardization for intrusion detection. The adaptive temperature mechanism adjusts soft-target smoothing according to training stage and sample difficulty, while per-sample Z-score logit standardization alleviates scale mismatch between teacher and student outputs. Building on these established mechanisms, we evaluate their combined use within the lightweight NIDS setting;
3. We evaluate the proposed method on NSL-KDD and CIC-IDS2017 from multiple perspectives, including convergence behavior, repeated-run stability, ablation analysis, efficiency indicators, statistical testing, difficulty-measure comparison, and lightweight baseline comparison. The results show that the distilled student maintains stable and competitive detection performance while substantially reducing model complexity, indicating a favorable accuracy–complexity trade-off for lightweight NIDS deployment;

The remainder of this paper is organized as follows. Section 2 reviews related work. Section 3 introduces CNN-based intrusion detection and knowledge distillation. Section 4 presents the proposed lightweight intrusion detection framework based on temperature-adaptive and logit-standardized distillation. Section 5 describes the experimental setup, datasets, and evaluation metrics. Section 6 reports and discusses the experimental results. Finally, Section 7 concludes this paper and summarizes the main contributions.

## 2 Related work

With the rapid evolution of edge computing and heterogeneous network environments, NIDS must achieve low-latency inference and deployment capabilities on resource-constrained endpoints and edge devices while maintaining high detection accuracy. This has spurred extensive research in model compression and lightweight design, where KD has gained widespread adoption due to its efficient teacher-pupil learning paradigm. The soft-target distillation framework proposed by Hinton et al. [7] employs a temperature parameter to smooth the output distribution, enabling the student model to capture relative relationships between classes. This approach reduces model complexity while preserving acceptable detection performance. Gou et al. [8] further highlighted that the selection of the temperature parameter, differences in teacher-student architectures, and training stability are critical factors determining distillation effectiveness.

Regarding temperature control and output-distribution modeling, conventional KD methods commonly treat the temperature as a global hyper-parameter, although adaptive temperature strategies have also been explored in recent studies. In particular, Li et al. [6] proposed Curriculum Temperature for Knowledge Distillation (CTKD), in which the temperature is treated as a learnable curriculum variable and optimized during training. In contrast, the temperature used in this study is explicitly determined according to the student’s predictive uncertainty and training progress rather than optimized as an additional learnable variable. A related line of research stems from model calibration: Guo et al. [9] show that temperature scaling can effectively mitigate over-confidence in deep networks, providing relevant background for temperature-based probability calibration. Furthermore, motivated by the scale mismatch between teacher and student logits, Sun et al. [10] proposed Logit Standardization to reduce the influence of logit-scale mismatch during distillation. The logit-standardization component adopted in this study follows this established formulation and is combined with the adaptive temperature schedule in the lightweight NIDS setting.

For lightweight NIDS applications, recent efforts have combined KD with feature learning and/or metric learning to better fit industrial and IoT scenarios. Wang et al. [11] propose a lightweight detection scheme for industrial cyber–physical systems (CPS) by integrating KD with metric-learning-oriented strategies to improve effectiveness under resource constraints. Another stream emphasizes the synergy between feature-domain transformation and distillation: Wang et al. [12] develop a lightweight intrusion detection model based on CNNs and KD, where frequency-domain or feature transformations are employed to enhance representation learning, and a compact student is trained via distillation to reduce inference overhead. Although these studies demonstrate the feasibility of KD for IoT/NIDS, they largely rely on heuristic temperature settings and standard distillation losses, lacking a unified modeling of adaptive temperature scheduling across training stages/classes and explicit alignment of logit distributions.

When data cannot be shared and client distributions are heterogeneous, combining federated learning (FL) with distillation provides a viable route for distributed NIDSs. DeepFed by Li et al. [13] demonstrates the effectiveness of federated training for intrusion detection in industrial CPS and offers a representative implementation paradigm for end–edge–cloud collaborative learning. To cope with stronger heterogeneity and privacy constraints, Li et al. [14] further propose a generative federated distillation framework for flow-level IoT intrusion detection, where a server-side generator assists client training to mitigate non-IID-induced performance degradation. Meanwhile, Shao et al. [15] highlight the engineering need for automated architecture search of lightweight federated models and propose Fed-GA-CNN-IDS to reduce model design and deployment costs. Nevertheless, these studies often emphasize robustness and architectural optimization in distributed training, while systematic and reproducible evaluations of single-model distillation stability (temperature and logit scale) and the trade-off between lightweight cost and detection performance remain limited.

Beyond model compression, class imbalance is another critical factor that affects the benefit of distillation in NIDSs: the scarcity of samples for many attack types makes the compressed student more prone to collapsing toward majority classes. Chen et al. [16] summarize the key challenges and strategies in imbalanced learning and emphasize the necessity of jointly suppressing majority-class bias from both data-level and algorithm-level perspectives. Yang et al. [17] further discuss the effectiveness of combining metric learning with oversampling mechanisms to alleviate imbalance, indicating that “representation-space restructuring + data re-balancing” is essential for stable minority-class recognition. Consistently, Mujahid et al. [18] conduct a systematic comparison of oversampling methods, including ADASYN, and demonstrate that properly designed resampling can significantly improve classification performance in imbalanced tasks.

In summary, existing studies indicate that knowledge distillation can effectively support lightweight deployment of NIDS and shows promising potential in federated and heterogeneous settings. Nevertheless, three aspects remain to be further strengthened. First, although adaptive temperature mechanisms have been explored in general knowledge distillation, their formulations and applications in lightweight NIDS remain relatively limited. Second, although logit standardization has been introduced in general knowledge distillation, its integration with adaptive temperature control has received limited attention in lightweight intrusion detection. Third, systematic and reproducible evaluation of the trade-off between lightweight indicators and detection performance is still underdeveloped. To further clarify the positioning of the present study, Table 1 compares the proposed method with representative recent KD-based and lightweight NIDS studies.

**Table 1:** Comparison with representative recent KD-based and lightweight NIDS studies.

Method	KD	Adaptive temp.	Logit align.	Efficiency eval.	Stat. valid.	Main distinction
[11]	✓	×	×	✓	×	KD + metric learning for industrial CPS
[12]	✓	×	×	✓	×	CNN-based lightweight NIDS with KD
[14]	✓	×	×	×	×	Generative federated distillation for IoT
[19]	✓	×	×	✓	×	Transformer-based KD with explainability
[20]	✓	×	×	✓	×	Dual-teacher KD for IIoT intrusion detection
Proposed method	✓	✓	✓	✓	✓	Adaptive temperature + logit standardization + multi-perspective evaluation

*Note:* KD denotes KD-based; Adaptive temp. denotes adaptive temperature; Logit align. denotes logit standardization or explicit logit alignment; Efficiency eval. denotes computational-efficiency evaluation; Stat. valid. denotes statistical validation. ✓ and × indicate the presence and absence of the corresponding item, respectively.

As shown in Table 1, the present study jointly incorporates adaptive temperature scheduling and logit standardization within a lightweight NIDS framework and evaluates the resulting configuration from multiple perspectives. Motivated by these observations, this work investigates the combined use of these mechanisms in lightweight intrusion detection and examines their behavior under the studied NIDS setting.

## 3 Theoretical background

### 3.1 Deep learning

Deep learning (DL), as a major branch of modern machine learning, is built upon multi-layer neural networks and achieves hierarchical data representation and automatic feature learning through successive nonlinear transformations [21]. Compared with traditional approaches that rely on handcrafted features, DL can directly extract high-level abstract features from raw data, substantially reducing the cost of feature engineering, and it typically exhibits stronger representational capacity and generalization ability in complex pattern recognition tasks.

In NIDSs, the objective is to identify potential malicious behaviors from massive, high-dimensional network traffic data or system logs, which entails challenges such as high feature dimensionality, diverse attack patterns, and temporal evolution of attacks. Owing to their strong capability in feature extraction and dependency modeling, deep learning models have become one of the mainstream approaches for building high-performance NIDSs [22]. In particular, CNNs are commonly adopted to capture local spatial characteristics in traffic data, whereas recurrent neural networks (RNNs) and their variants are used to model temporal dependencies of attack behaviors. Meanwhile, KD provides an effective means to compress deep models and reduce inference overhead while maintaining detection performance [7].

### 3.2 CNN

Convolutional neural networks are a representative class of deep learning models that were initially developed primarily for image processing. Inspired by the local receptive field mechanism of the human visual cortex, CNNs exhibit strong capability in extracting local features. A typical CNN consists of convolutional layers, nonlinear activation layers, pooling layers, and a small number of fully connected layers. Specifically, convolutional layers apply sliding kernels over the input to perform local perception with weight sharing, thereby extracting regional features; pooling layers reduce feature dimensionality and computational complexity while preserving salient information; and fully connected layers map high-level features to the final classification outputs [23]. Benefiting from local connectivity and weight sharing, CNNs can effectively reduce the number of parameters and improve training efficiency without sacrificing representational power. A schematic of the basic CNN architecture is illustrated in Fig 1.

**Fig 1:**
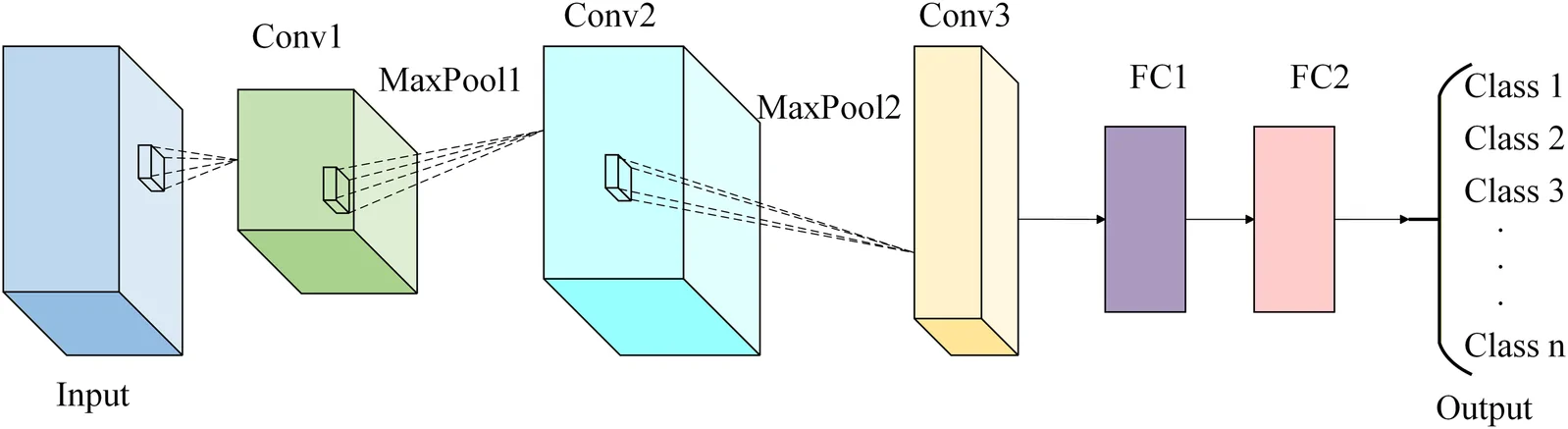
CNN architecture for classification.

In network intrusion detection systems, data such as network traffic or host logs can be reconstructed into matrix- or tensor-form inputs to CNNs. Through stacked convolutional operations, CNNs can automatically learn local discrepancies and pattern variations between benign behaviors and diverse attack behaviors, thereby enabling effective detection of potential malicious activities [24]. A large body of studies has shown that, compared with traditional methods relying on handcrafted features, CNN-based intrusion detection models generally achieve better overall performance in terms of metrics such as accuracy, recall, and F1-score, and they are particularly suitable for high-dimensional and multi-class attack scenarios [25,26]. In this work, the CNN architecture serves as the backbone network for both the teacher and student models to extract spatial features from network traffic, providing the representational basis for subsequent knowledge distillation and model lightweighting.

### 3.3 Knowledge distillation

Knowledge distillation is a machine learning technique that transfers the knowledge learned by a large pre-trained teacher model to a more compact student model. The concept was introduced by Hinton et al. [7] in 2015. Its key idea is to train the student to match the teacher’s output distribution (logits). Compared with the original 0–1 hard labels, the logit-based soft output distribution encodes richer inter-class relational information, which can improve the student’s generalization capability. As a representative paradigm of model compression and knowledge transfer in deep learning, KD is particularly suitable for large-scale deep neural networks. Under the teacher-student formulation, the student mimics the teacher’s output distribution to inherit its inference behavior and generalization ability, thereby improving student performance while substantially reducing computational requirements during deployment, which is beneficial for resource-constrained scenarios. A typical teacher-student framework is illustrated in Fig 2.

**Fig 2:**
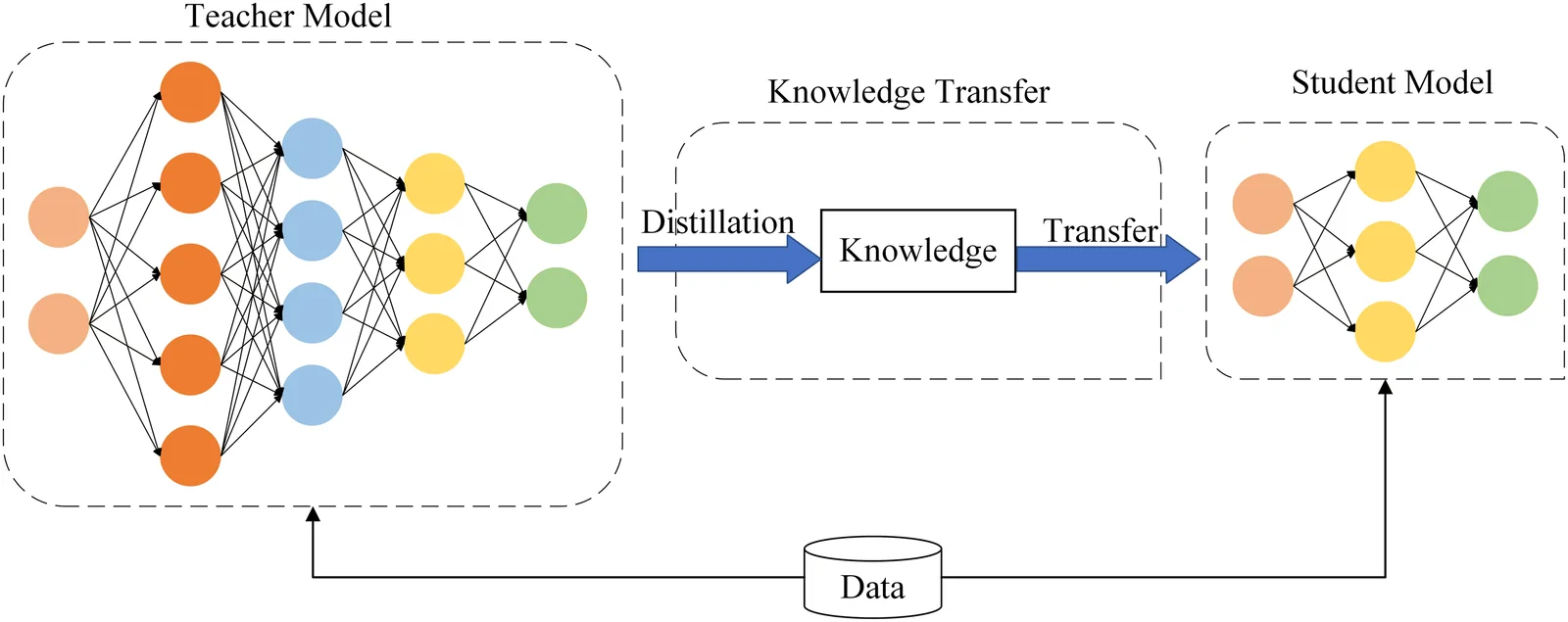
Schematic Diagram of a Typical Teacher-Student Structure.

From the perspective of knowledge types, KD is commonly categorized into three classes: response-based knowledge, feature-based knowledge, and relation-based knowledge [8], as illustrated in Fig 3. Response-based distillation directly uses the class-probability distribution at the teacher’s output layer as the learning target, where the student mimics the teacher’s predictions by minimizing the discrepancy between distributions. Feature-based distillation treats intermediate-layer representations of the teacher as the distillation targets and aligns the student’s representation space via feature matching or reconstruction losses [27]. Relation-based distillation further focuses on relational structures among samples or features across channels/layers, such as preserving relative distances or similarity structures induced by the teacher. Fig 4 presents an illustrative process of feature-based distillation.

**Fig 3:**
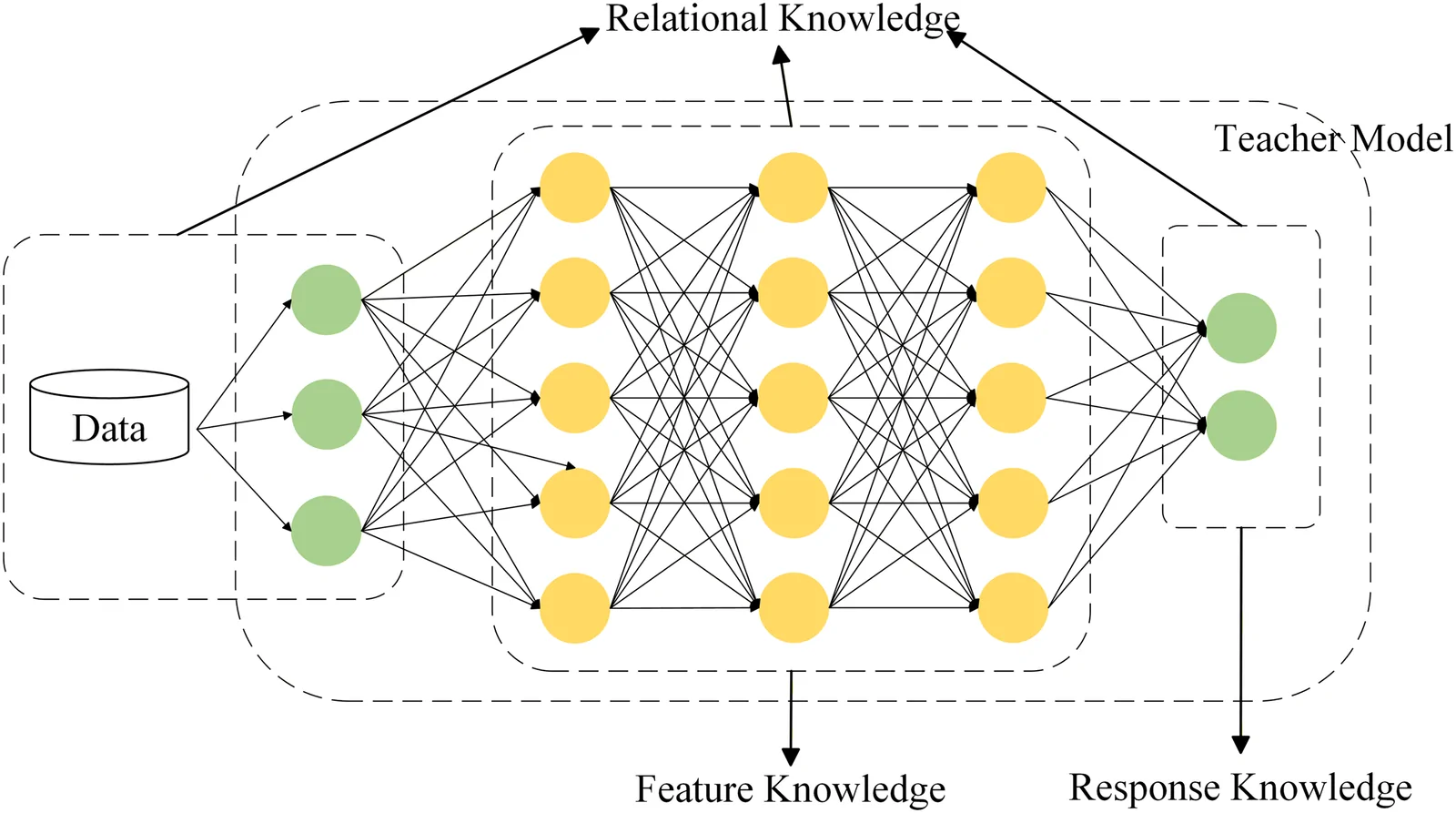
Schematic Diagram of Knowledge Types in the Teacher Model.

**Fig 4:**
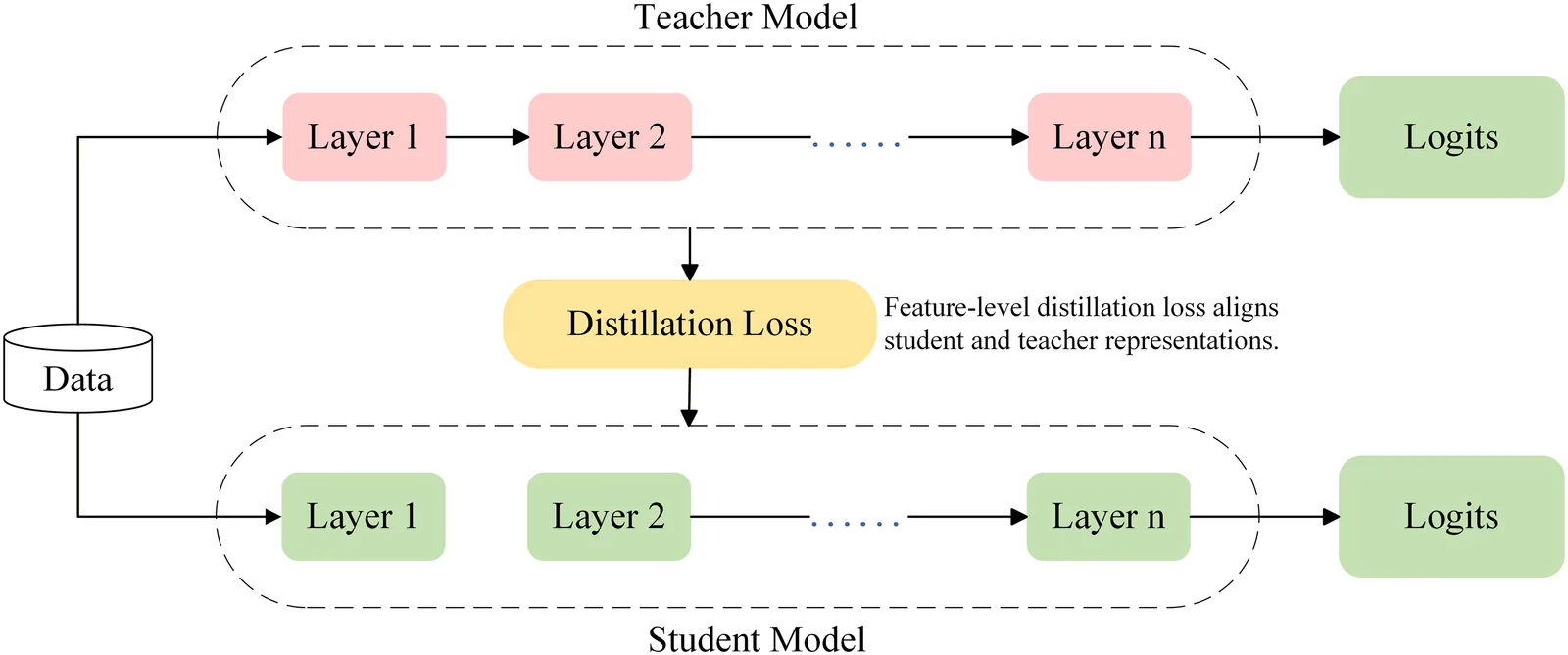
Feature-based knowledge distillation process.

Depending on the training procedure and the update strategy of teacher and student networks, KD can be broadly divided into offline distillation, online distillation, and self-distillation. Offline distillation is the canonical setting: a high-capacity teacher model is first trained independently; during distillation, the teacher is kept fixed and only the student is updated, such that the student is supervised by hard labels while being encouraged to match the teacher’s output distribution. This setting is straightforward to implement and typically exhibits good stability, and thus has been widely adopted. Online distillation updates the teacher and student simultaneously during training, which is often used when a strong pre-trained teacher is unavailable or when multiple networks are expected to improve collaboratively. Self-distillation can be viewed as a special case of online distillation, where the teacher and student share the same architecture, and “self-teaching” is achieved via different branches or training stages, improving a single network without introducing an additional large teacher model.

In intrusion detection, although complex deep models often achieve strong detection accuracy, real-time performance and deployability may become practical bottlenecks. KD provides a direct solution: a high-performing deep network is typically adopted as the teacher in the offline stage and trained on sufficiently collected data to obtain accurate modeling; during deployment, a distilled lightweight student model is used for real-time intrusion detection [19,20,28]. This strategy enables the student to inherit the teacher’s detection capability while substantially reducing computation and memory overhead at inference time. Building upon the classical temperature-based distillation framework, this work further incorporates Logit Standardization and an adaptive temperature mechanism to fine-tune the scale and smoothness of soft targets during distillation, thereby improving training stability and discriminative performance of lightweight intrusion detection models under complex and imbalanced data conditions.For clarity, the main notations used in the proposed method are summarized in Table 2.

**Table 2:** Main notations used in the proposed method.

Symbol	Meaning
$M_{T}$	Teacher model
$M_{S}$	Student model
$z_{T}$	Teacher logits
$z_{S}$	Student logits
*T*_0_	Initial upper bound of the adaptive temperature
$T_{t}(x)$	Adaptive temperature for sample *x* at training stage *t*
$\gamma_{t}$	Training progress factor
ω(x)	Uncertainty-based coefficient of sample *x*
*H*(*x*)	Predictive entropy of sample *x*
*q*(*T*)	Teacher predictive distribution under temperature scaling
*p*(*T*)	Student predictive distribution under temperature scaling

## 4 Proposed method

### 4.1 Adaptive temperature knowledge distillation

Knowledge distillation (KD) constructs a high-capacity teacher model and a compact student model, and constrains the student’s predictive distribution by leveraging the teacher’s soft labels and/or intermediate feature representations. In this way, KD aims to preserve the teacher’s discriminative capability while substantially reducing the parameter size and computational overhead of the deployed model. Compared with supervised learning that relies solely on ground-truth labels, soft labels explicitly encode relative inter-class similarities, which is particularly important for NIDS tasks that are multi-class, highly imbalanced, and characterized by complex attack patterns.

Motivated by this, we build a teacher–student architecture for lightweight NIDS and further incorporate an adaptive temperature scheduling strategy together with Logit Standardization into the classical temperature-based distillation framework. These two components address different aspects of distillation: adaptive temperature controls the degree of soft-target smoothing, while Logit Standardization is used to reduce logit-scale mismatch between teacher and student outputs. The overall framework is illustrated in Fig 5.

**Fig 5:**
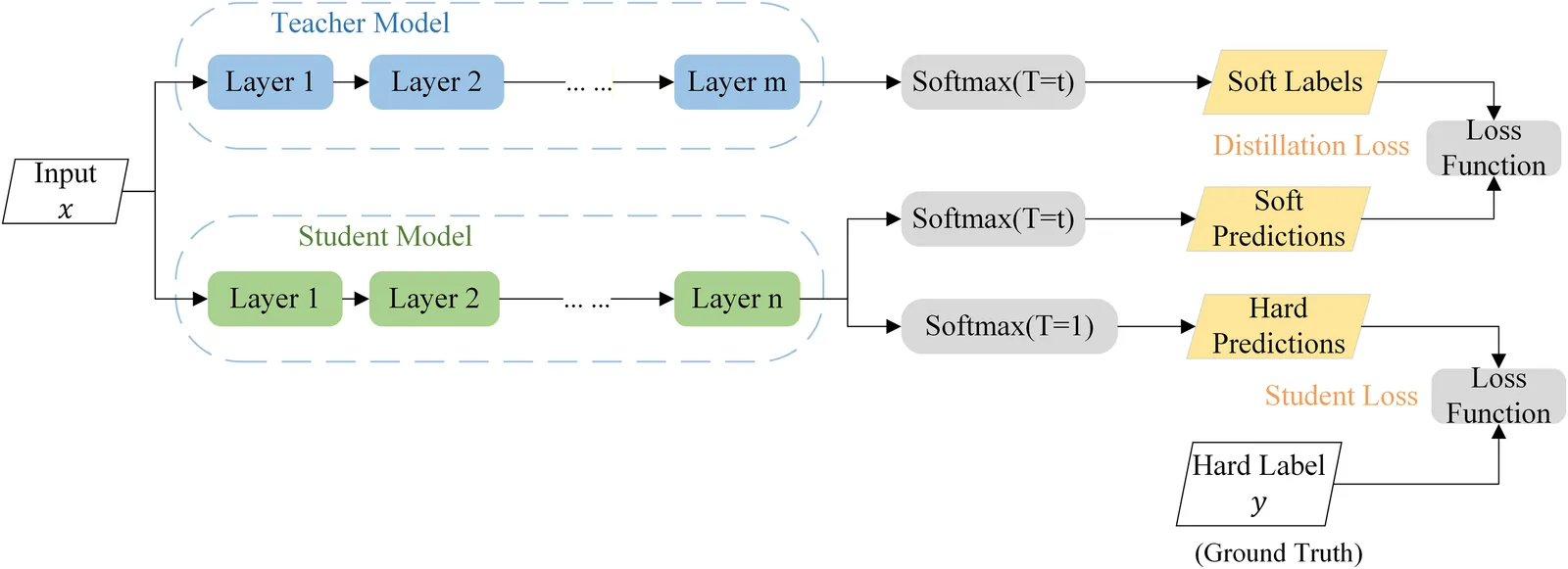
Temperature-adaptive KD framework.

During distillation, we introduce a temperature parameter *T* to scale the logits of both the teacher and the student. The temperature-scaled softmax is given by


$$\begin{array}{c} q_{i}(T)=\frac{\exp(z_{i}/T)}{\sum_{j=1}^{K}\exp(z_{j}/T)} \end{array}$$ (1)

where $z_{i}$ denotes the logit of class *i*, and $q_{i}(T)$ denotes the predicted probability under temperature *T*. When *T* > 1, the output distribution becomes smoother and the relative relationships among classes are emphasized; when *T* approaches 1, the output distribution becomes sharper and less smoothed.

Unlike conventional approaches using a fixed temperature, we dynamically adapt the temperature by jointly considering predictive uncertainty and training stage, thereby providing more appropriate distillation signals for different samples and different training stages. Specifically, we first quantify predictive uncertainty using predictive entropy. Let $p(x)=Softmax(z_{S}(x))$ denote the current student’s predictive distribution at *T* = 1. The predictive entropy is defined as


$$\begin{array}{c} H(x)=-\sum_{i=1}^{K}p_{i}(x)\log p_{i}(x) \end{array}$$ (2)

A larger entropy indicates higher predictive uncertainty of the current student model, which is used as an uncertainty indicator for adaptive temperature adjustment rather than an absolute measure of sample difficulty. We then construct a coefficient ω(x) that is monotonically increasing with *H*(*x*). To ensure consistent scaling across different numbers of classes *K*, we use the normalized form


$$\begin{array}{c} \omega (x)=\frac{H(x)}{\log K},\;\omega (x)\in [0,1] \end{array}$$ (3)

In addition, we introduce a training progress factor $\gamma_{t}\in [0,1]$ to model the global adjustment at training stage *t*: it takes a larger value in early training *t*o emphasize the “dark knowledge” relationships among classes, and gradually decreases as training proceeds to strengthen fine-grained discrimination around the decision boundary. By combining predictive uncertainty and training progress, and to guarantee T≥1 with an upper bound controlled by the initial setting, we define the adaptive temperature as


$$\begin{array}{c} T_{t}(x)=1+(T_{0}-1)\cdot \gamma_{t}\cdot \omega (x),\;T_{0}>1 \end{array}$$ (4)

Since $\gamma_{t}$ and ω(x) are both normalized within the range of [0,1], the adaptive temperature is constrained within [1,*T*_0_]. This bounded formulation limits excessive temperature variation during training.

Accordingly, samples with higher predictive uncertainty and early training stages receive larger temperatures, producing smoother soft targets that facilitate knowledge transfer from the teacher. As training progresses and predictive uncertainty decreases, the temperature is gradually reduced, producing sharper and less-smoothed soft targets.

The proposed temperature-adaptive KD objective consists of a soft distillation loss and a hard supervised loss, formulated as


$$\begin{array}{c} ℒ=\alpha \;T_{t}(x{)}^{2}\;ℋ\;\left(q(T_{t}),p(T_{t})\right)+\beta \;ℋ(y,p), \end{array}$$ (5)

where ℋ(·,·) denotes the cross-entropy function, $q(T_{t})$ and $p(T_{t})$ denote the teacher and student predictive distributions under temperature $T_{t}$, respectively, *y* is the ground-truth label, and α and β are weighting coefficients. The first term is the temperature-scaled soft distillation loss that encourages the student to match the teacher’s soft-target distribution, while the second term is the hard-label loss that ensures discriminative accuracy on the target classes. With the joint optimization of adaptive temperature scheduling and soft-hard supervision, the student is encouraged to balance knowledge transfer from the teacher model and predictive accuracy on the target classes while maintaining a lightweight architecture.

### 4.2 Logit standardization in knowledge distillation

In conventional KD, the teacher and the student typically share the same temperature parameter. Although this setting is straightforward to implement, previous studies have shown that mismatched logit scales between teacher and student models may affect distribution alignment during distillation. To alleviate this issue, Logit Standardization has been introduced as an effective mechanism for normalizing logit distributions. In this study, we incorporate this established mechanism into the lightweight NIDS distillation framework.

#### 4.2.1 Logit shift.

Under the shared-temperature setting, let $z_{S,n}$ denote the student logit vector for the *n*-th sample and $z_{T,n}$ denote the corresponding teacher logit vector. Their means are defined as


$$\begin{array}{c} {\bar{z}}_{S,n}=\frac{1}{K}\sum_{k=1}^{K}z_{S,n,k},\;{\bar{z}}_{T,n}=\frac{1}{K}\sum_{k=1}^{K}z_{T,n,k}, \end{array}$$ (6)

where $z_{S,n,k}$ and $z_{T,n,k}$ represent the logits of class *k* for the *n*-th sample from the student and the teacher, respectively, and *K* is the number of classes. Since softmax is invariant to a global translation, when the optimization is mainly driven by distribution matching, there can be a shift ambiguity between teacher and student logits: the two logit vectors may differ by an additive constant while yielding the same softmax distribution. With shared temperature and direct distribution alignment, such ambiguity may introduce additional coupling between teacher and student logit scales. This coupling can influence the optimization process, especially when there is a large capacity gap between teacher and student models.

#### 4.2.2 Variance matching.

Moreover, the temperature coefficient can be viewed as a factor that controls the scale of logits. Under this perspective, the dispersion of student and teacher logits (measured by variance) satisfies


$$\begin{array}{c} \frac{\sigma^{2}(z_{S,n})}{\sigma^{2}(z_{T,n})}=\frac{\frac{1}{K}\sum_{k=1}^{K}{\left(z_{S,n,k}-{\bar{z}}_{S,n}\right)}^{2}}{\frac{1}{K}\sum_{k=1}^{K}{\left(z_{T,n,k}-{\bar{z}}_{T,n}\right)}^{2}}=\frac{b_{S}^{2}}{b_{T}^{2}}. \end{array}$$ (7)

where $\sigma^{2}(z_{S,n})$ and $\sigma^{2}(z_{T,n})$ denote the variances of the student and teacher logits, respectively, and $b_{S}$ and $b_{T}$ are the temperature coefficients for the student and the teacher. When the temperature is shared, $b_{S}=b_{T}$, the above reduces to


$$\begin{array}{c} \sigma (z_{S,n})=\sigma (z_{T,n}) \end{array}$$ (8)

This implies that, under shared temperature and distribution alignment, the logit scale of the student may be implicitly coupled to that of the teacher, resulting in a certain degree of “variance matching”. For a low-capacity student, such implicit coupling may reduce the flexibility of adjusting its output scale during distillation.

#### 4.2.3 Logit standardization and adaptive temperature scaling.

To alleviate the above potential constraints, we apply sample-wise Logit Standardization to both teacher and student logits before temperature scaling. Consistent with the adaptive-temperature formulation in Eq. 4, the standardized teacher and student logits share the same sample-dependent temperature $T_{t}(x)$ during distillation.

For the *n*-th sample, the student and teacher logits are standardized as


$$\begin{array}{c} z_{S,n,k}^{\prime }=\frac{z_{S,n,k}-\mu (z_{S,n})}{\sigma (z_{S,n})},\;z_{T,n,k}^{\prime }=\frac{z_{T,n,k}-\mu (z_{T,n})}{\sigma (z_{T,n})}. \end{array}$$ (9)

This sample-wise normalization transforms both logit vectors into zero-mean, unit-variance representations, reducing the influence of absolute logit scale while preserving relative inter-class relationships.

The standardized logits are then scaled by the same adaptive temperature $T_{t}(x)$ and passed through softmax:


$$\begin{array}{c} q(T_{t})=Softmax\left(\frac{z_{T}^{\prime }}{T_{t}(x)}\right),\;p(T_{t})=Softmax\left(\frac{z_{S}^{\prime }}{T_{t}(x)}\right). \end{array}$$ (10)

The resulting teacher and student distributions are used to compute the soft distillation loss in Eq. 5. Under this formulation, Logit Standardization reduces teacher–student logit-scale mismatch, whereas the adaptive temperature controls the degree of soft-target smoothing.

### 4.3 Overall training algorithm

In NIDSs, model lightweighting is essential to address the deployment difficulty of conventional deep models on resource-constrained devices, which is primarily caused by their large parameter sizes and high computational overhead. In this work, we employ KD together with Logit Standardization to build a lightweight yet high-performance NIDS model. The core objective of lightweighting is not only to reduce the number of parameters, but also to reduce computational cost and support efficient inference in resource-limited scenarios such as edge computing. The lightweighting workflow is illustrated in Fig 6.

**Fig 6:**
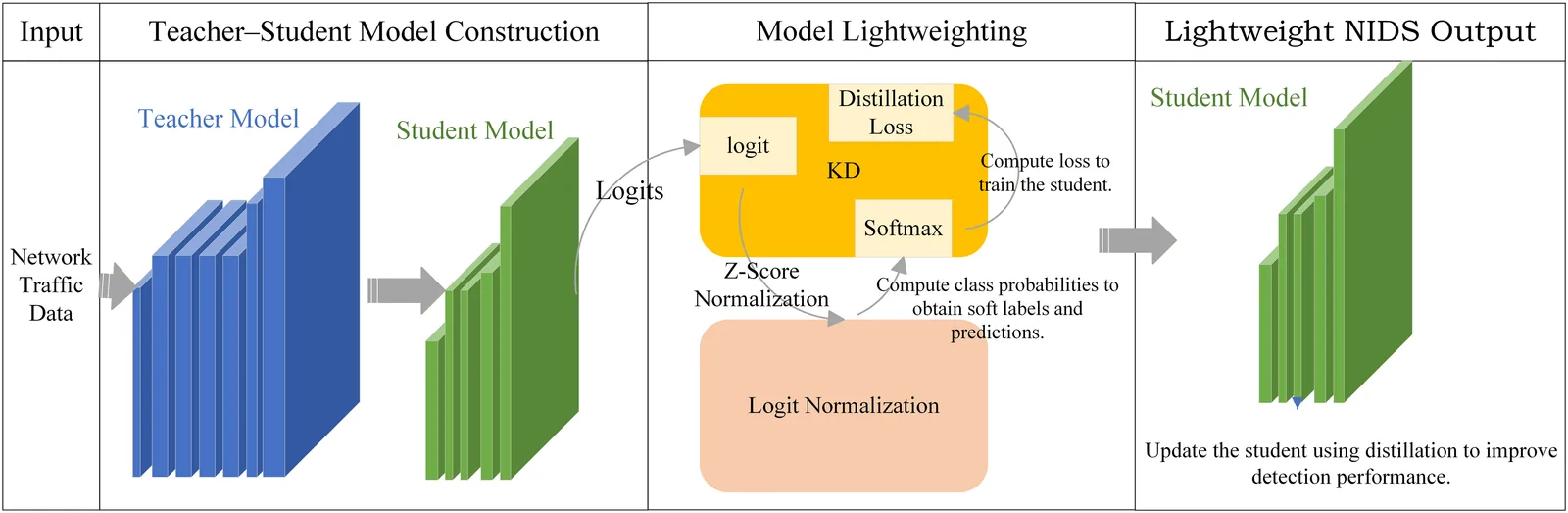
KD workflow for lightweight NIDS.

In KD, the architectures of the teacher and student models substantially affect both the lightweighting gain and the efficiency of knowledge transfer. As shown in Fig 7, the teacher model adopts a relatively complex architecture that includes InceptionModule, Conv1d, AdaptiveMaxPool1d, BatchNorm1d, BiGRU, and MultiheadAttention modules. It produces higher-dimensional representations and contains more parameters, thereby providing strong feature extraction and classification capability. In contrast, the student model follows the same modular design principle but uses reduced hyper-parameter configurations, e.g., fewer convolution channels, smaller hidden dimensions in BiGRU, and lower embedding dimensions in MultiheadAttention, which reduces the parameter size and computational cost to support lightweight inference.

**Fig 7:**
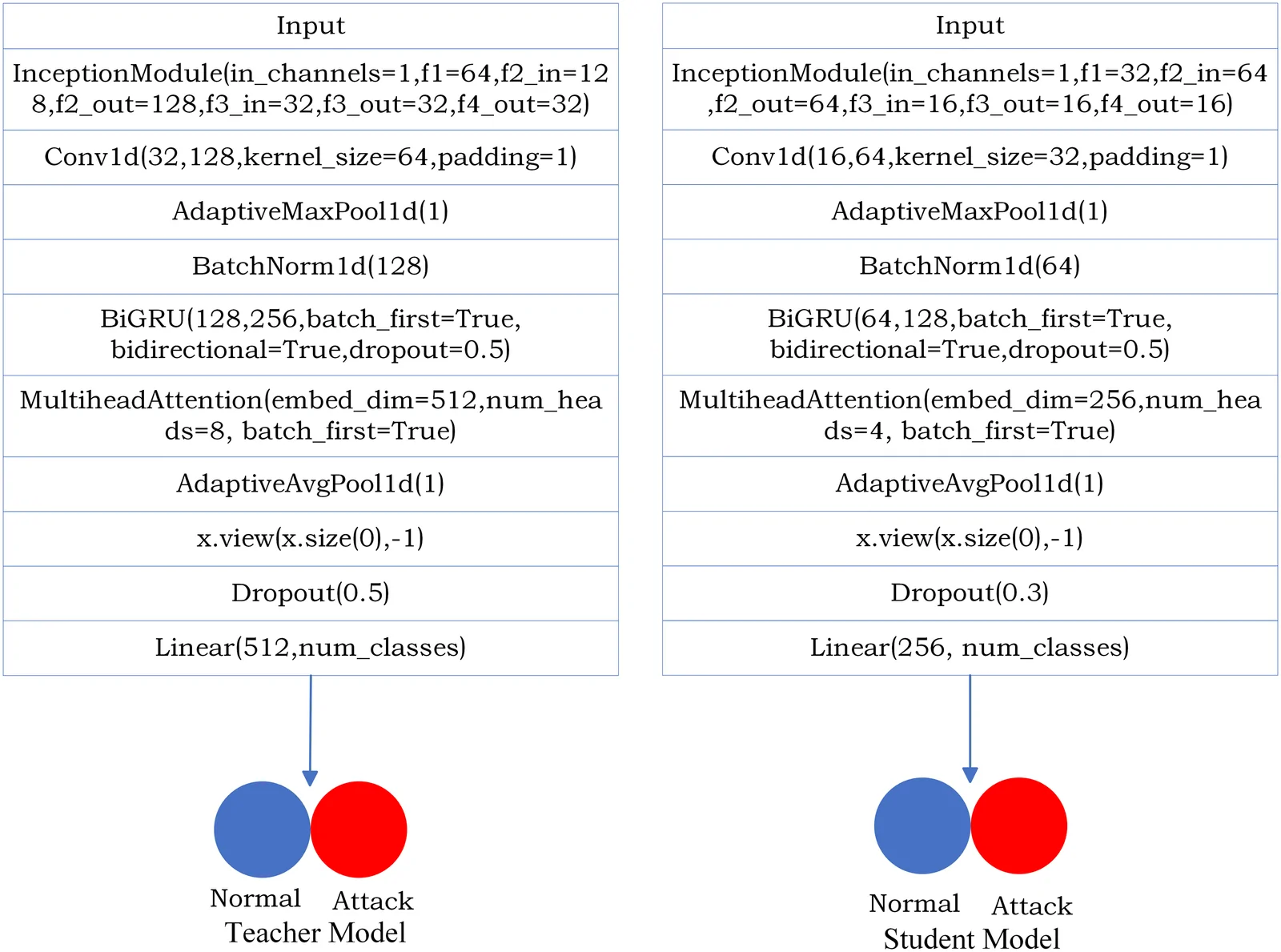
Teacher–student architecture comparison.

In conventional KD, the student is often implicitly encouraged to match the teacher in terms of the numerical range and variance of logits, which introduces additional fitting pressure for a capacity-limited lightweight student. To alleviate this issue, we incorporate Logit Standardization into the distillation branch and jointly apply it with temperature-adaptive distillation. Specifically, the logits of both the teacher and the student are standardized on a per-sample basis via Z-score normalization:


$$\begin{array}{c} z^{\prime }=\frac{z-\mu (z)}{\sigma (z)} \end{array}$$ (11)

where *z* denotes the original logits, μ(z) and σ(z) are the mean and standard deviation of the sample-wise logits, respectively, and $z^{\prime }$ denotes the standardized logits. This operation reduces the impact of absolute scale discrepancy on the distillation loss and encourages the student to focus on relative class ordering and decision-structure information. Moreover, since Z-score normalization is a linear transformation, the relative ordering of logits remains unchanged, which facilitates the transfer of priority information among critical classes.

With the temperature-adaptive mechanism, the standardized logits from the teacher and the student are further processed via temperature scaling and softmax to produce soft targets, which are jointly used with ground-truth labels to supervise the student training. For reproducibility and future extensions, we formalize the overall training procedure as Algorithm 1, which systematically describes the teacher–student interactions across training stages and the integrated pipeline of “dynamic temperature + Logit Standardization.”

**Algorithm 1. Temperature-Adaptive and Logit-Standardized Knowledge Distillation for Lightweighting**

Input: Teacher model $M_{T}$ (frozen), student model $M_{S}$, training set *D*_train_, initial temperature upper bound *T*_0_ > 1, training progress coefficient $\gamma_{t}\in [0,1]$, loss weights α,β, maximum epochs *E*, number of classes *K*

Output: Trained lightweight student model $M_{S}^{*}$

1:  Randomly initialize student parameters $M_{S}$

2:  Load teacher parameters $M_{T}$ (pre-trained; frozen; no backprop)

3:  Set temperature upper bound *T*_0_

4:  **for** *e* = 1 **to** *E* **do**

5:   **for** each mini-batch $(X,y)\in D_{train}$ **do**

6:    $z_{T}\leftarrow M_{T}(X)$                    // teacher logits

7:    $z_{S}\leftarrow M_{S}(X)$                       // student logits

8:    $p\leftarrow Softmax(z_{S})$                     // hard branch (*T* = 1)

9:    $H(X)\leftarrow -\sum_{i=1}^{K}p_{i}(X)\log p_{i}(X)$                // entropy; vectorizable

10:    ω(X)←H(X)/logK                   // normalize to [0,1]

11:    $T(X)\leftarrow 1+(T_{0}-1)\cdot \gamma_{t}\cdot \omega (X)$ // T(X)≥1, $\leq T_{0}$

12:    $z_{T}^{\prime }\leftarrow \frac{z_{T}-\mu (z_{T})}{\sigma (z_{T})}$                     // logit standardization

13:    $z_{S}^{\prime }\leftarrow \frac{z_{S}-\mu (z_{S})}{\sigma (z_{S})}$

14:    $q(T)\leftarrow Softmax(z_{T}^{\prime }/T(X))$                 // teacher soft labels

15:    $p(T)\leftarrow Softmax(z_{S}^{\prime }/T(X))$                 // student soft preds

16:    $L_{soft}\leftarrow ℋ(q(T),p(T))$

17:    $L_{hard}\leftarrow ℋ(y,p)$

18:    $L\leftarrow \alpha \;T(X{)}^{2}\;L_{soft}+\beta \;L_{hard}$

19:    Update $M_{S}$ by backpropagation using ∇L

20:   **end for**

21:  **end for**

22:  **return** $M_{S}^{*}$

As shown in Algorithm 1, the student model is jointly supervised by the teacher-provided soft targets and the ground-truth labels during training. With the aid of Logit Standardization and adaptive temperature scheduling, the student is able to better capture global inter-class relationships in the early training stage and gradually enhance fine-grained discriminative capability in the later stage.

## 5 Experimental environment setup

### 5.1 Preparation for experiment

In Table 3 below, the experimental environment for this study is depicted.

**Table 3:** Experimental environment.

Module	Parameter
Operating System	Windows 11
CPU	Intel(R) Core(TM) i7-14700HX
GPU	NVIDIA GeForce RTX 4060 Laptop GPU
RAM	80.00 GB
Software	Python 3.9; Matplotlib 3.7.2; PyTorch 1.13.0 + cu117

### 5.2 Model configuration and training settings

To improve reproducibility, the model configuration and training settings used in this study are summarized as follows. The proposed lightweight intrusion detection framework adopts a teacher–student distillation setting, in which the teacher model is first pre-trained and then kept frozen during student training, while the student model is randomly initialized and optimized through backpropagation. Unless otherwise stated, all compared methods are trained under a unified protocol with a batch size of 256, an initial learning rate of $5\times 10^{-4}$, a weight decay coefficient of $1\times 10^{-5}$, and a maximum of 30 training epochs. For the proposed distillation framework, the soft distillation loss weight is set to α=0.7, and the upper bound of the adaptive temperature is set to *T*_0_ = 5.0. For the repeated-run experiments, each configuration is executed with five random seeds, namely 42, 52, 62, 72, and 82, and the results are reported as mean ± standard deviation. Unless otherwise specified, all compared methods are trained under the same protocol, and Adam is used as the optimizer.

### 5.3 Evaluation metrics

To comprehensively evaluate model performance, four representative metrics are adopted in this work, including accuracy (ACC), precision (Pre), recall (Rec), and F1-score. These metrics are derived from the evaluation matrix constructed based on the predicted labels and the ground-truth labels, which serves as the basis for quantitative evaluation.

The entries in the confusion matrix are defined as follows:TP (True Positive): the number of attack samples correctly classified as attacks, reflecting the model’s ability to identify the positive class (attacks).TN (True Negative): the number of benign samples correctly classified as benign, indicating the model’s ability to recognize the negative class (normal/benign).FP (False Positive): the number of benign samples incorrectly classified as attacks. A high FP leads to excessive false alarms and increases the investigation workload, degrading practical usability.FN (False Negative): the number of attack samples incorrectly classified as benign. A high FN indicates severe missed detections, which may result in potential security risks being overlooked.

Accuracy measures the proportion of correctly classified samples among all samples, computed as


$$\begin{array}{c} Accuracy=\frac{TP+TN}{TP+TN+FP+FN} \end{array}$$ (12)

While accuracy reflects overall correctness, it can be misleading under class-imbalanced settings.

Precision measures the proportion of true positives among samples predicted as positive, given by


$$\begin{array}{c} Precision=\frac{TP}{TP+FP} \end{array}$$ (13)

Recall measures the proportion of true positives among all actual positive samples, computed as


$$\begin{array}{c} Recall=\frac{TP}{TP+FN} \end{array}$$ (14)

F1-score is the harmonic mean of precision and recall, capturing their balance, and is computed as


$$\begin{array}{c} F1\text{-}score=\frac{2\times Precision\times Recall}{Precision+Recall} \end{array}$$ (15)

The F1-score alleviates the one-sidedness of using precision or recall alone and is often more informative under class-imbalanced scenarios.For multi-class intrusion detection tasks, Macro-F1 and Weighted-F1 are further reported as complementary evaluation metrics. Macro-F1 is the arithmetic mean of the F1-scores over all classes and emphasizes the balanced performance of the model across different categories. In contrast, Weighted-F1 computes the weighted average of class-wise F1-scores according to the number of samples in each class, and thus better reflects the overall classification performance under imbalanced class distributions.

In addition to detection metrics, computational efficiency indicators are also considered. Specifically, Params denotes the total number of trainable parameters in the model, reflecting model size and storage requirements, while FLOPs denotes the theoretical number of floating-point operations required for one forward inference pass, reflecting computational complexity. These indicators are used to compare the computational efficiency and lightweight characteristics of different models.

## 6 Results

### 6.1 Simulation experiments

To examine the optimization behavior and stability of the proposed lightweight distillation framework for network intrusion detection, this study first analyzes the convergence behavior of the distilled student model on two public datasets, namely NSL-KDD and CIC-IDS2017. Fig 8a–8d present the training and validation accuracy curves together with the corresponding loss curves on the two datasets. Overall, the model converges rapidly in the early training stage on both datasets and remains in a high-performance region during later epochs, indicating stable optimization behavior of the distilled student model under the lightweight setting.

**Fig 8:**
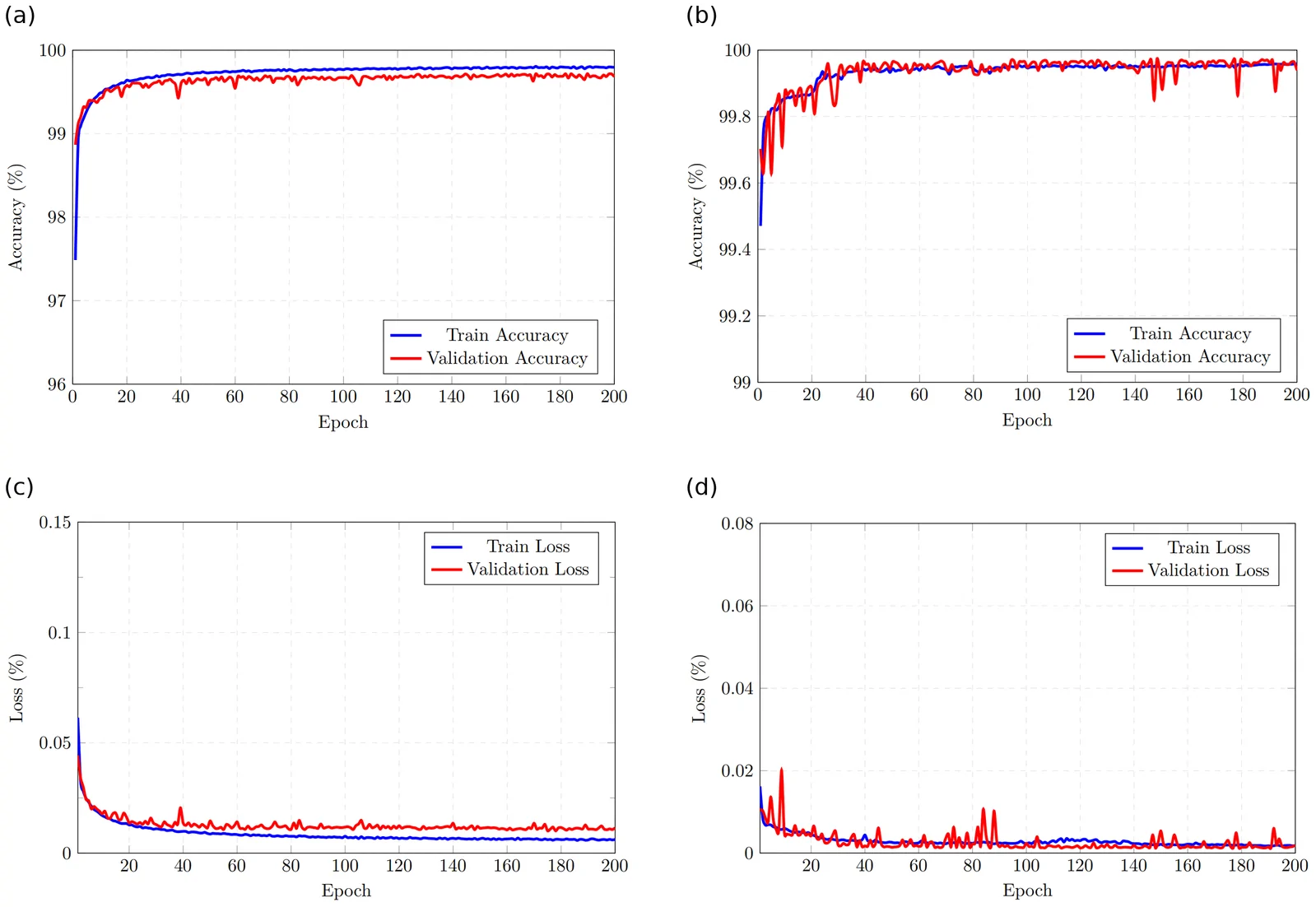
Convergence curves of the distilled student model on NSL-KDD and CIC-IDS2017. (a) Training and validation accuracy on NSL-KDD. (b) Training and validation accuracy on CIC-IDS2017. (c) Training and validation loss on NSL-KDD. (d) Training and validation loss on CIC-IDS2017.

Fig 8 shows that, on NSL-KDD, the training and validation accuracy curves are highly consistent, while the loss curves decrease rapidly and gradually stabilize, indicating good convergence behavior and fitting performance on this dataset. By contrast, the accuracy and loss curves on CIC-IDS2017 exhibit more noticeable fluctuations in later epochs, although the model still maintains high accuracy and low loss overall. This suggests that the distilled student model remains reasonably stable on the more diverse CIC-IDS2017 traffic distribution. To further assess this behavior across multiple independent runs, repeated-run experiments were conducted on both datasets. Table 4 reports the mean ± standard deviation of Accuracy, Macro-F1, and Weighted-F1 for different methods on NSL-KDD and CIC-IDS2017.

**Table 4:** Repeated-run results.

Method	Dataset	Accuracy	Macro-F1	Weighted-F1
Student only	NSL-KDD	99.54 ± 0.03	99.54 ± 0.03	99.54 ± 0.03
	CIC-IDS2017	99.88 ± 0.01	82.13 ± 0.67	99.88 ± 0.01
Std KD	NSL-KDD	99.57 ± 0.03	99.57 ± 0.03	99.57 ± 0.03
	CIC-IDS2017	99.89 ± 0.01	82.05 ± 0.76	99.88 ± 0.01
Adaptive T	NSL-KDD	99.55 ± 0.02	99.55 ± 0.02	99.55 ± 0.02
	CIC-IDS2017	99.84 ± 0.10	81.09 ± 5.98	99.84 ± 0.10
Logit Std	NSL-KDD	99.56 ± 0.03	99.56 ± 0.03	99.56 ± 0.03
	CIC-IDS2017	99.89 ± 0.01	83.15 ± 2.14	99.89 ± 0.01
Full Model	NSL-KDD	99.56 ± 0.01	99.56 ± 0.01	99.56 ± 0.01
	CIC-IDS2017	99.89 ± 0.01	81.24 ± 1.71	99.88 ± 0.01

*Note:* Student only denotes supervised training with the student model only; Std KD denotes standard knowledge distillation; Adaptive T denotes knowledge distillation with adaptive temperature; Logit Std denotes knowledge distillation with logit standardization; Full Model denotes the complete method combining adaptive temperature and logit standardization.

Table 4 shows that, on NSL-KDD, Std KD, Logit Std, and Full Model achieve closely comparable mean performance, while Full Model exhibits relatively smaller standard deviations, indicating relatively stable run-to-run behavior while maintaining comparable performance. On CIC-IDS2017, the differences among the methods in Accuracy and Weighted-F1 are generally limited, whereas Logit Std performs relatively better in terms of Macro-F1. Overall, the complete framework demonstrates stable performance under the repeated-run setting.

### 6.2 Ablation and efficiency analysis

To further analyze the proposed method, this study compares the training behavior of the teacher model and the distilled student model, and analyzes the contribution of different components from the perspectives of ablation results and computational efficiency indicators. Fig 9a–9d illustrate the training accuracy and loss curves of the teacher and student models on NSL-KDD and CIC-IDS2017.

**Fig 9:**
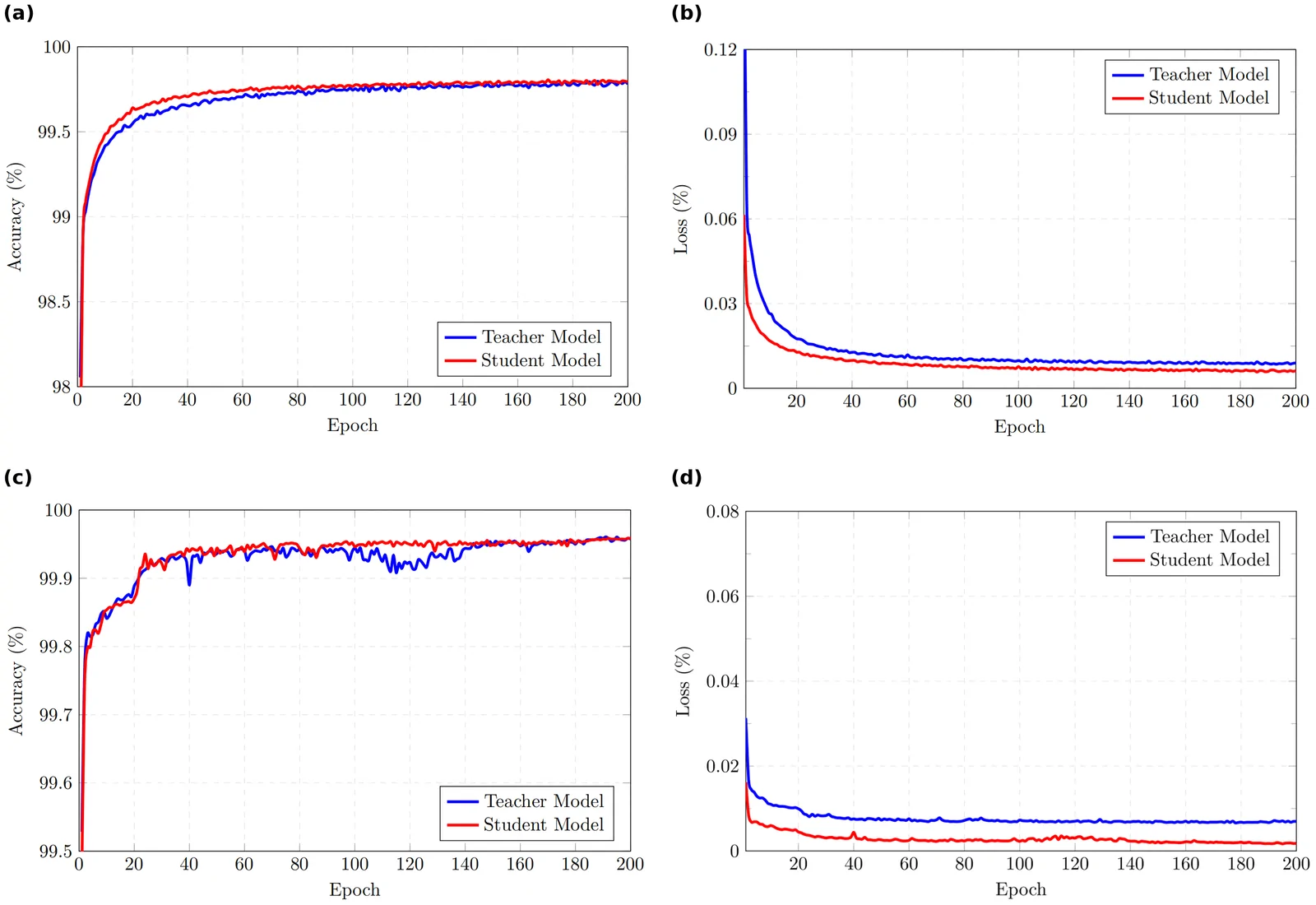
Teacher–student convergence comparison before and after lightweighting on NSL-KDD and CIC-IDS2017. (a) Teacher vs. student training accuracy on NSL-KDD. (b) Teacher vs. student training loss on NSL-KDD. (c) Teacher vs. student training accuracy on CIC-IDS2017. (d) Teacher vs. student training loss on CIC-IDS2017.

As shown in Fig 9, on NSL-KDD, both the teacher and student models exhibit rapidly increasing training accuracy in the early stage and stabilize at a high level in later epochs. The corresponding loss curves also decrease quickly and remain close to each other. On CIC-IDS2017, the two models show similar overall convergence trends. Although the student model exhibits some fluctuations during part of the training process, it eventually converges to a comparable range. These observations indicate that the distilled student maintains good convergence behavior and training stability on both datasets. To quantitatively examine the role of different distillation components, Table 5 reports the ablation results on NSL-KDD and CIC-IDS2017.

**Table 5:** Ablation study results.

Method	Dataset	Accuracy	Macro-F1	Weighted-F1	CPU Latency	GPU Latency
					(ms)	(ms)
Student only	NSL-KDD	99.54	99.54	99.54	1.9200	1.3400
	CIC-IDS2017	99.90	83.16	99.90	1.8035	1.3691
Std KD	NSL-KDD	99.59	99.59	99.59	1.7040	1.3280
	CIC-IDS2017	99.89	81.79	99.89	1.8320	1.3542
Adaptive T	NSL-KDD	99.56	99.56	99.56	1.9240	1.3280
	CIC-IDS2017	99.89	84.22	99.89	1.9859	1.3717
Logit Std	NSL-KDD	99.59	99.59	99.59	1.7530	1.3420
	CIC-IDS2017	99.89	81.86	99.89	1.9037	1.3605
Full Model	NSL-KDD	99.55	99.55	99.55	1.7470	1.3440
	CIC-IDS2017	99.89	81.80	99.89	1.7992	1.3456

Table 5 indicates that, on NSL-KDD, Std KD and Logit Std achieve relatively stronger overall performance, while Adaptive T also remains competitive. Full Model is close to these stronger baselines but does not consistently lead across all metrics. This suggests that adaptive temperature scheduling and logit standardization affect different aspects of the distillation process, while their combination does not yield a simple additive gain. Specifically, adaptive temperature controls soft-target smoothing, whereas logit standardization reduces logit-scale mismatch during distribution alignment. On CIC-IDS2017, the differences among the distillation variants in Accuracy and Weighted-F1 are generally small, while Adaptive T yields the highest Macro-F1 in this ablation setting, indicating that temperature adaptation alone can also be competitive under some settings. To further analyze computational efficiency, Table 6 presents comparisons of parameter count, model size, FLOPs, CPU latency, and GPU latency.

**Table 6:** Model complexity and inference timing.

Method	Dataset	Params	Model Size	FLOPs	CPU Latency	GPU Latency
			(MB)		(ms)	(ms)
Teacher	NSL-KDD	1,970,821	7.531	39,137,536	1.695	1.423
	CIC-IDS2017	1,972,360	7.537	25,861,632	2.210	1.563
Student	NSL-KDD	462,149	1.775	5,895,296	1.562	1.357
	CIC-IDS2017	462,920	1.778	3,917,568	1.789	1.461
Tiny Student	NSL-KDD	112,549	0.441	991,552	1.886	1.311
	CIC-IDS2017	112,936	0.443	663,936	1.933	1.356
Dynamic Quant Student	NSL-KDD	311,872	1.351	5,743,232	1.215	—
	CIC-IDS2017	311,872	1.352	3,764,736	1.190	—

*Note:* Teacher denotes the original high-capacity teacher model; Student denotes the distilled baseline student model; Tiny Student denotes a further compressed smaller student variant; Dynamic Quant Student denotes the dynamically quantized student model.

From Table 6, compared with the Teacher model, the Student model substantially reduces the number of parameters, model size, and theoretical computational complexity on both datasets, while also showing lower CPU and GPU latency under the evaluated hardware setting. Further comparison shows that Tiny Student provides stronger structural compression, whereas Dynamic Quant Student achieves lower CPU latency.

### 6.3 Extended Performance Analysis

In addition to overall convergence and average performance, class-level behavior is also important for evaluating lightweight intrusion detection models. Therefore, this study further compares the class-wise predictions of the Teacher and Student models based on the confusion matrices on NSL-KDD and CIC-IDS2017. Figs 10 and 11 present the confusion matrices on the two datasets, respectively.

**Fig 10:**
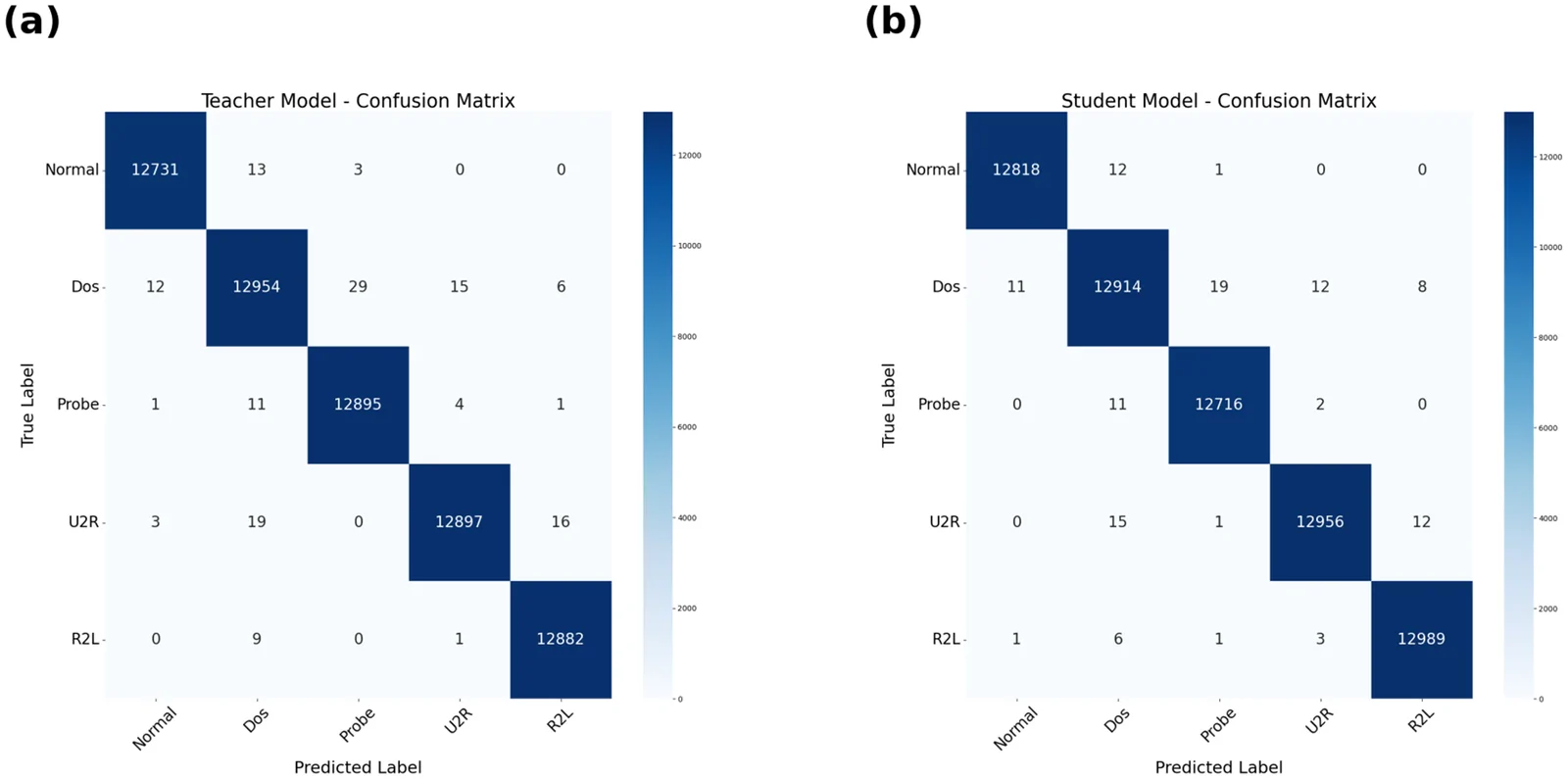
Confusion matrices on NSL-KDD. (a) Teacher model. (b) Distilled lightweight student model.

**Fig 11:**
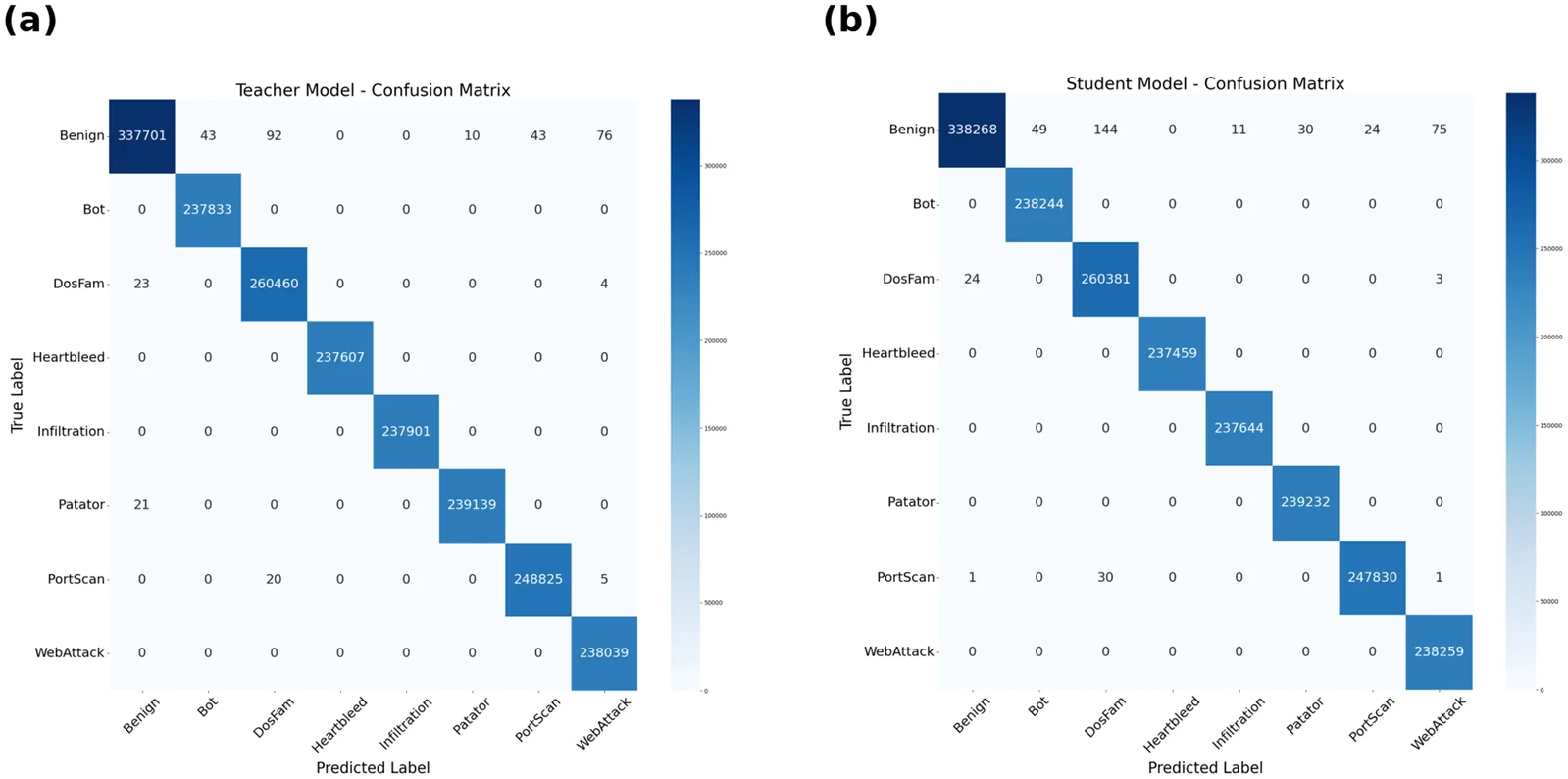
Confusion matrices on CIC-IDS 2017. (a) Teacher model. (b) Distilled lightweight student model.

From Fig 10, it can be seen that on NSL-KDD, the Student model largely preserves the discriminative capability of the Teacher model for the major attack categories. For most classes, the predictions of the Student model remain highly consistent with those of the Teacher model, while more misclassifications mainly occur for a few complex classes. This suggests that the lightweighting process largely preserves the main discriminative structure of the teacher model, although some information loss still exists in fine-grained class discrimination.

Fig 11 shows that on CIC-IDS2017, the Student model likewise preserves the recognition ability for major traffic classes and representative attack types. Overall, the confusion distributions of the Student and Teacher models are similar, although some gaps remain for more complex or less represented attack categories. Taken together, the results on the two datasets indicate that the distilled student retains the main discriminative capability of the teacher model while maintaining a compact structure. To further examine the statistical characteristics of performance differences among different methods, Table 7 reports the statistical significance test results of Full Model against Std KD and Student only on NSL-KDD and CIC-IDS2017.

**Table 7:** Statistical significance test results.

Comparison	Metric	Dataset	Paired	Paired	Wilcoxon	Wilcoxon
			t-stat	t-p	stat	p
Full Model vs Std KD	Accuracy	NSL-KDD	−0.6896	0.5284	6	0.8125
		CIC-IDS2017	0.1170	0.9125	7	1.0000
	Weighted-F1	NSL-KDD	−0.6768	0.5356	6	0.8125
		CIC-IDS2017	0.1352	0.8990	6	0.8125
	Macro-F1	NSL-KDD	−0.6731	0.5378	6	0.8125
		CIC-IDS2017	−0.8451	0.4456	6	0.8125
Full Model vs Student only	Accuracy	NSL-KDD	1.3038	0.2623	3	0.3125
		CIC-IDS2017	2.1655	0.0963	2	0.1875
	Weighted-F1	NSL-KDD	1.3069	0.2613	3	0.3125
		CIC-IDS2017	2.1385	0.0993	2	0.1875
	Macro-F1	NSL-KDD	1.3146	0.2589	3	0.3125
		CIC-IDS2017	−1.0721	0.3441	6	0.8125

*Note:* Paired t-stat and Wilcoxon stat denote the test statistics of the paired t-test and Wilcoxon signed-rank test, respectively; Paired t-p and Wilcoxon p denote the corresponding p-values for assessing statistical significance.

From Table 7, all paired t-test p-values and Wilcoxon p-values are greater than 0.05, indicating that the performance differences between Full Model and the two compared baselines do not reach statistical significance under the current repeated-run setting. These results suggest that, under the current repeated-run setting, the observed differences should be interpreted as indicative rather than conclusive. Combined with the repeated-run results in Table 4, Full Model maintains relatively stable performance on both datasets. On this basis, to further compare the effect of different difficulty measures, Table 8 reports the results of three difficulty measures, namely Entropy, Margin, and Loss, on NSL-KDD and CIC-IDS2017.

**Table 8:** Comparison of difficulty measures.

Difficulty Type	Dataset	Accuracy	Macro-F1	Weighted-F1
entropy	NSL-KDD	99.51	99.51	99.51
margin		99.53	99.53	99.53
loss		99.56	99.56	99.56
entropy	CIC-IDS2017	99.89	82.78	99.89
margin		99.89	82.45	99.89
loss		99.89	81.84	99.88

From Table 8, the effectiveness of different difficulty measures exhibits clear dataset dependence. On NSL-KDD, loss-based difficulty achieves the best results in Accuracy, Macro-F1, and Weighted-F1, followed by margin-based difficulty, while entropy-based difficulty is slightly lower. On CIC-IDS2017, entropy- and margin-based difficulty achieve comparable Accuracy and Weighted-F1, whereas entropy-based difficulty performs better in Macro-F1. These results suggest that different difficulty measures exhibit dataset-dependent behavior under the evaluated settings. Furthermore, to compare the proposed method with other lightweight strategies, Table 9 reports the results of Full Model and several lightweight baselines, including Student only, Tiny Student, Pruned Student (30%), and Dynamic Quant Student.

**Table 9:** Comparison of lightweight baselines.

Method	Dataset	Accuracy	Macro-F1	Weighted-F1	Params	FLOPs	CPU	GPU
							Latency	Latency
							(ms)	(ms)
Student only	NSL-KDD	99.54	99.54	99.54	462,149	5,895,296	1.6842	1.3447
	CIC-IDS2017	99.89	82.92	99.89	462,920	3,917,568	2.0020	1.3950
Tiny Student	NSL-KDD	99.41	99.41	99.41	112,549	991,552	1.9222	1.3123
	CIC-IDS2017	99.89	83.89	99.88	112,936	663,936	2.0570	1.4040
Pruned Student (30%)	NSL-KDD	99.52	99.52	99.52	462,149	5,895,296	1.9467	1.3557
	CIC-IDS2017	99.89	81.79	99.89	462,920	3,917,568	1.7640	2.0830
Dynamic Quant Student	NSL-KDD	99.54	99.54	99.54	311,872	5,743,232	1.3697	—
	CIC-IDS2017	99.89	82.92	99.89	311,872	3,764,736	1.6090	—
Full Model	NSL-KDD	99.55	99.55	99.55	462,149	5,895,296	1.7981	1.3267
	CIC-IDS2017	99.89	84.02	99.89	462,920	3,917,568	2.0680	1.4090

*Note:* Student only denotes supervised training with the student model only; Tiny Student denotes a further compressed smaller student variant; Pruned Student (30%) denotes the student model after 30% pruning; Dynamic Quant Student denotes the dynamically quantized student model; Full Model denotes the complete method combining adaptive temperature and logit standardization.

From Table 9, on NSL-KDD, the classification performance of Full Model is close to that of the stronger baselines, while on CIC-IDS2017, its Macro-F1 is relatively better. Meanwhile, different methods exhibit different trade-offs among structural compression, computational cost, and latency. Tiny Student emphasizes model compression, Dynamic Quant Student achieves lower CPU latency, and Pruned Student does not consistently show stronger overall classification performance. Overall, Full Model maintains a relatively balanced trade-off between classification performance and lightweight efficiency.

## 7 Conclusion

This study investigates a lightweight network intrusion detection approach based on knowledge distillation, integrating a temperature-adaptive distillation schedule with logit standardization to study their combined effect under resource-constrained settings. Specifically, we apply per-sample Z-score standardization to both teacher and student logits to reduce scale mismatch, allowing distillation to emphasize relative inter-class relationships during distribution alignment.

Experimental results on NSL-KDD and CIC-IDS2017 indicate that the distilled student maintains stable and competitive detection performance while following the teacher’s overall convergence trends. The student substantially reduces model overhead, with approximately 76.5% fewer parameters and substantially lower theoretical FLOPs. In addition to the convergence comparison, the repeated-run, ablation, efficiency, and lightweight baseline analyses further show that the proposed framework achieves a favorable accuracy–complexity trade-off for lightweight intrusion detection. The current statistical testing results also indicate that the performance differences between Full Model and the two compared baselines do not reach statistical significance under the present experimental setting. Therefore, the main value of the proposed method lies in its stable overall performance, lightweight efficiency, and favorable accuracy–complexity trade-off rather than uniform superiority across all compared settings.

The efficiency analysis in this study is based on model complexity and inference timing under the evaluated hardware setting, while real deployment performance still requires further validation on dedicated edge devices. Future work will focus on real-device deployment validation, direct quantitative comparison with recent adaptive KD methods such as CTKD under the same NIDS setting, comparison with recent lightweight methods, and further refinement of adaptive distillation strategies to improve the practical applicability of lightweight network intrusion detection.
